# Impact of *MIF* Gene Promoter Polymorphism on F508del Cystic Fibrosis Patients

**DOI:** 10.1371/journal.pone.0114274

**Published:** 2014-12-12

**Authors:** Paola Melotti, Andrea Mafficini, Patrick Lebecque, Myriam Ortombina, Teresinha Leal, Emily Pintani, Xavier Pepermans, Claudio Sorio, Baroukh Maurice Assael

**Affiliations:** 1 Cystic Fibrosis Centre, University and Hospital Trust of Verona, Verona, Italy; 2 ARC-NET Research Centre and Department of Pathology and Diagnostics, University and Hospital Trust of Verona, Verona, Italy; 3 Pediatric Pulmonology & Cystic Fibrosis Unit, Université Catholique de Louvain, Brussels, Belgium; 4 Louvain Centre for Toxicology and Applied Pharmacology, Université Catholique de Louvain, Brussels, Belgium; 5 Centre for Human Genetics; Université Catholique de Louvain, Brussels, Belgium; University of Thessaly, Faculty of Medicine, Greece

## Abstract

Macrophage migration Inhibitory Factor (MIF) is a pro-inflammatory cytokine sustaining the acute response to gram–negative bacteria and a regulatory role for MIF in Cystic Fibrosis has been suggested by the presence of a functional, polymorphic, four-nucleotide repeat in this gene's promoter at position −794, with the 5-repeat allele displaying lower promoter activity. We aimed at assessing the association of this polymorphism with disease severity in a group of Cystic Fibrosis patients homozygous for F508del *CFTR* gene mutation. Genotype frequencies were determined in 189 Cystic Fibrosis and 134 control subjects; key clinical features of patients were recorded and compared among homozygous 5-allele patients and the other *MIF* genotypes. Patients homozygous for the 5-repeat allele of *MIF* promoter displayed a slower rate of lung function decline (p = 0.027) at multivariate survival analysis. Multiple regression analysis on age-normalized respiratory volume showed no association of the homozygous 5-repeat genotype with lung function under stable conditions and no correlation with *P.aeruginosa* chronic colonization. Therefore, only the Homozygous 5-repeat genotype at *MIF* −794 is associated with milder disease in F508del Cystic Fibrosis patients.

## Introduction

Cystic fibrosis (CF) is the most common, severe, inherited disorder in the Caucasian population. It is caused by mutations in the CF Transmembrane conductance Regulator (*CFTR*) gene and mainly characterized by bronchopulmonary disease, pancreatic insufficiency and male infertility. Patients with identical *CFTR* genotypes can display markedly different phenotypic expression [1], [2] and modifier genes were previously described among the factors causing this discrepancy [3], [4]. Macrophage Migration Inhibitory Factor (MIF) is a key pro-inflammatory mediator [5]: it sustains an acute inflammatory response both directly, by inducing cytokines secretion, and indirectly, by overriding the anti-inflammatory activity of glucocorticoids [6]. MIF plays a significant role in immune and inflammatory-based diseases such as asthma [7], rheumatoid arthritis [8], acute respiratory distress syndrome [9] and septic shock [10], [11]. Although MIF is involved in the defence against severe infection, modulation of the high cytokine levels elicited by its action may prevent harmful effects during the inflammatory response. Indeed, lethal sepsis induced in mice by lipopolysaccharide (LPS) or *E. coli* causes increased mortality in the presence of recombinant MIF [12], while anti–MIF neutralizing antibodies were able to protect mice from lethal endotoxic sepsis induced by bacterial (*E. coli*) peritonitis [11]. It has also been suggested that neutralizing MIF could lead to improved resistance against *P. aeruginosa* infection, since clearance of the bacteria following tracheal instillation was improved in MIF-knockout mice [10]. Recently, Baugh et al. [13] identified a functionally significant polymorphism in the human MIF gene, consisting of a four-nucleotide CATT repeat located at position −794 of the MIF promoter (MIF-CATT). In an *in vitro* model, the 5-CATT repeat showed significantly lower transcriptional activity when compared to the 6-, 7- or 8-CATT repeat alleles. This polymorphism is reported as a TTCA insertion or deletion relative to the 6-repeats genotype in NCBI dbSNP entries rs3063368 and rs36224313 respectively, at the genomic coordinates (UCSC genome browser – hg19) chr22:24235773-24235772. Five percent of healthy subjects are homozygous for the 5-CATT repeat allele. Homozygosity for this allele was significantly associated with milder forms of rheumatoid arthritis, suggesting it may have a protective effect. In CF patients, Plant et al. [14] reported a significant decrease in both *P. aeruginosa* colonization and pancreatic insufficiency among adult patients carrying at least one 5-CATT *MIF* allele. Since many studies of modifier genes in CF have yielded conflicting results, it is essential to validate any association in a new, independent population and *MIF* gene is no exception [4]. This study aimed at clarifying and validating the association between MIF-CATT repeats and disease severity in a more homogeneous cohort of CF patients with homozygous F508del CFTR mutation. Given the biological relationship between MIF and acute inflammation suggested by the above cited literature, we chose as a primary outcome the time to the first acute episode causing forced expiratory volume (FEV1) to fall below the 60% of the predicted value. We also verified the possible relationship between MIF and age-normalized FEV1 and chronic *P. aeruginosa* colonization under stable conditions.

## Materials and Methods

### Study population

One hundred and eighty-nine CF patients homozygous for the F508del mutation were recruited from two European centres (Verona: 138, Brussels: 51). All of the patients were able to perform reliable spirometry. A cohort of 134 adult Italian subjects was used as control. Healthy subjects were negative for the most common mutations of the *CFTR* gene, except for 4 heterozygous subjects (healthy carriers). This is consistent with epidemiological data about carrier frequency in Europe. This study was approved by the Ethics Committee of the University and Hospital Trust of Verona (protocol #24737); informed signed consent for DNA analysis was obtained from participants or from their parents, as required.

### Genotyping

DNA was extracted from whole blood using the salting out method, then samples were genotyped for the polymorphism of *MIF* promoter (varying number of CATT repeats) at -794. DNA was amplified by Polymerase Chain Reaction (PCR) in a GeneAmp PCRsystem 9700® (Applied Biosystem, Foster City, CA, USA) as previously described [13]. Genotyping was performed by the BMR Genomics Sequencing Service (CRIBI, University of Padova, Italy). Results were analysed using GenescanView 1.2 software (CRIBI, University of Padova, Italy).

### Clinical data of CF patients

Clinical data for the 189 patients were collected in electronic databases. Main characteristics and *MIF* genotype of the patients are summarized in **table 1** and extensively reported in S1 Table. Anthropometric parameters and forced expiratory volume (FEV1) were normalized using Freeman's [15] and Knudson's [16] equations respectively. Given the accelerated FEV1 decline with age in CF patients, the last value of FEV1 was expressed as FEV1 percentile using CF-specific reference equations [17]. Diabetes was defined by the need for insulin therapy. Chronic *P. aeruginosa* colonization was reported using a European consensus definition [18]. In brief, chronic colonization was defined as the isolation of at least 3 isolates in a six month period (at minimum 30 days interval) while sporadic colonization referred to the isolation of *P. aeruginosa* in the bronchial tree in presence or absence of inflammation.

**Table 1:** Characteristics of 189 Cystic Fibrosis patients homozygous for the F508del mutation recruited from 2 different European centers.

	Brussels	Verona	p-value
**Subjects n**	51	138	
**Female n (%)**	22 (%)	78 (%)	0.14
**Age, years**	21.5±9.4	24.27±9.3	0.09
**FEV1, Kulich** *	67±24	45±31	<0.0001
**BMI z-score**	−0.63±1.03	−0.82±1.28	0.36
**cc by PA n (%)**	16 (31.4%)	87 (63.0%)	<0.0002
**Diabetes n (%)**	11 (21.6%)	40 (29.0%)	0.36
**MIF-CATT 5-5 n (%)**	4 (7.8%)	12 (8.7%)	
**MIF-CATT 5-6 n (%)**	12 (23.5%)	59 (42.8%)	
**MIF-CATT 5-7 n (%)**	7 (13.7%)	10 (7.2%)	0.11
**MIF-CATT 6-6 n (%)**	20 (39.2%)	43 (31.2%)	
**MIF-CATT 6-7 n (%)**	8 (15.7%)	12 (8.7%)	
**MIF-CATT 7-7 n (%)**	0 (0%)	2 (1.4%)	

Continuous data are presented as mean ± SD unless otherwise stated; categorical data are presented as counts and proportions. FEV1: forced expiratory volume in one second; cc by PA: chronic colonization by *P. aeruginosa.* The most recent FEV1 was used for each patient. MIF-CATT: *MIF* gene -CATT repeat genotype at position -794

* CF specific percentile according to Kulich *et al*, *Am J Respir Crit Care Med*, 2005.

### Statistical analysis and study design

Given the involvement of MIF in acute inflammation, we focused on the FEV1 parameter as primary outcome variable, comparing patients with the 5-5 MIF genotype to the rest of the cohort. The relationship between FEV1 and MIF genotype was analysed considering both the time to the first acute episode causing a FEV1 value under 60%, and the “static” FEV1 at last visit (normalised according to Kulich's CF-specific reference equations [17]).

Considering the differences in clinical parameters between patients from the two centres (table 1), the analyses were performed using multivariate techniques. Cox regression was used to analyse the time to first FEV1 under 60% and patient's origin (Verona or Brussels) was included as a covariate together with presence of *MIF* 5-5 genotype. Chronic colonization by *P. aeruginosa* and presence of insulin-dependent diabetes were not considered because only a minority of patients displayed these features before first acute episode (28.9% for *P.aeruginosa* and 5.0% for diabetes). Given the unequal size of 5-5 and X-X groups (patients number ratio  = 0.09), we used the R package PowerSurvEpi to calculate the power to detect an effect like the one estimated from our data (Hazard ratio  = 0.32) with the number of patients available. The power resulted to be 59%, while the sample size needed to get 80% power would have been 307 patients. Multiple linear regression was used to analyse last visit FEV1, including as covariates *MIF* 5-5 genotype, patients' origin, chronic colonization by *P. aeruginosa* and presence of insulin-dependent diabetes; age was not included because FEV1 values were already age-normalized according to Kulich. Variance inflation factor was used to monitor the presence of multicollinearity; its value was below 1.15 for all covariates.

Power analysis for last visit FEV1 was also used to calculate the power to detect a medium effect size (0.5, here corresponding to a difference between means of 15%, which was similar to the estimated difference in our cohort) in an unpaired t-test with our sample size and considering the unequal size of 5-5 (n = 16, mean FEV1 = 50.2, SD = 31.3) and X-X groups (n = 173, mean FEV1 = 64.2, SD = 25.5). The power calculated was 48%, while the sample size needed to get 80% power would have been 382 patients. All analyses were performed using GraphPad Prism version 5.04 for Windows (GraphPad Software, San Diego California USA, www.graphpad.com), MedCalc for Windows, version 14.8.1 (MedCalc Software, Ostend, Belgium) and the R software (R Development Core Team, version 2.9; R Foundation for Statistical Computing, Vienna, Austria; www.R-project.org). Power analysis for multiple linear regression was performed with G*Power [19].

## Results

### Genotyping of the −794 CATT polymorphic repeats of *MIF* promoter (MIF-CATT)

In 189 CF patients, frequencies for 5, 6, 7 and 8 MIF-CATT repeats were 31.7%, 57.4%, 10.9% and 0% respectively. Corresponding values for the 135 healthy control subjects were similar (28.5%, 60%, 11.1% and 0.4%). In CF patients, MIF-CATT genotype frequencies were similar among children and adults, excluding a survival bias for 5-5 subjects.

### Clinical status of CF patients and MIF-CATT genotype

Patients were categorized according to the −794 CATT polymorphic repeats (MIF-CATT). Anthropometric and clinical data are summarized in **Table 1** **and** **2**, and extensively reported in S1 Table.

**Table 2:** Clinical data of 187 Cystic Fibrosis patients homozygous for the F508del mutation according to the genotype for the *MIF* gene -CATT repeat at position −794.

	5–5	5–6	5–7	6–6	6–7
n	16	71	17	63	20
**Age, years (95% CI)**	18.2 (14.5– 21.9)	23.6 (21.3–25.9)	27.2 (23.1– 31.3)	23.7 (21.2–26.1)	22.3 (18.2–26.5)
**BMI z-score (95% CI)**	−0.51 (−1.10/0.08)	−0.84 (−1.17/−0.52)	−1.09 (−1.63/−0.54)	−0.71 (−0.97/−0.46)	−0.62 (−1.26/0.01)
**FEV1, Kulich** * **(95% CI)**	64.2 (50.6–77.8)	43.9 (36.6–51.2)	52.4 (37.7–67.08)	53.8 (45.7–61.8)	59.3 (44.8–73.8)
**CC by PA – n (%; 95% CI)**	5 (31.2%; 13.9–55.8%)	44 (62.0%; 50.3–72.4%)	11 (64.7%; 41.2–82.8%)	33 (52.4%; 40.3–64.2%)	8 (40.0%; 21.8–61.4%)
**Diabetes – n (%; 95% CI)**	2 (12.5%; 2.2–37.3%)	22 (31.0%; 21.4–42.5%)	6 (35.3%; 17.2–58.8%)	17 (27.0%; 17.5–39.1)	4 (20.0%; 7.5–42.2%)

Data are presented as mean and 95% Confidence Interval (95% CI). FEV1: forced expiratory volume in one second; BMI: body mass index; cc by PA: chronic colonization by *P. aeruginosa*.

* CF specific percentile according to Kulich *et al*, *Am J Respir Crit Care Med*, 2005.

Given the involvement of MIF in acute inflammation, we focused on FEV1 as primary outcome variable, comparing patients with the 5-5 MIF-CATT genotype to the rest of the cohort.

The relationship between FEV1 and MIF genotype was analysed considering both the time to the first acute episode causing a FEV1 value under 60% and, secondly, the “static” FEV1 at last visit. Considering the differences in clinical parameters between patients from the two centres and the possible effect of diabetes and *P. aeruginosa* colonization on pulmonary volume, the following analyses were performed using multivariate techniques.

### MIF-CATT 5-5 genotype is associated to a later onset of acute episodes

Cox regression was used to analyse the time to the first acute episode causing FEV1 to fall under 60% predicted. MIF-CATT 5-5 genotype and patient's origin (Verona or Brussels) were included as independent variables. Chronic colonization by *P. aeruginosa* and presence of insulin-dependent diabetes were not considered because only a minority of patients displayed these features before first acute episode (28.9% for *P.aeruginosa* and 5.0% for diabetes). Both MIF-CATT 5-5 genotype (Hazard Ratio = 0.327; 95% CI 0.121-0.884) and belonging to the Brussels cohort (Hazard Ratio = 0.514; 95% CI 0.310 to 0.849) resulted to be independent predictors of a later onset of acute episodes (**Table 3**).

**Table 3:** Cox regression analysis for age at first acute episode with FEV1 <60% of predicted value on 185 Cystic Fibrosis patients homozygous for the F508del mutation.

Variable	Value	Hazard Ratio	95% CI	p-value
MIF-CATT genotype	X-X	1	-	-
	5–5	0.325	0.120–0.878	0.027
Centre of origin	Verona	1	-	-
	Brussels	0.510	0.309–0.843	0.0090

Number of patients with acute episode  = 119

Number of censored patients = 66

Overall significance p-value  = 0.0010

MIF-CATT genotype: *MIF* gene -CATT repeat genotype at position −794

95% CI  = 95% Confidence Interval

**Figure 1:**
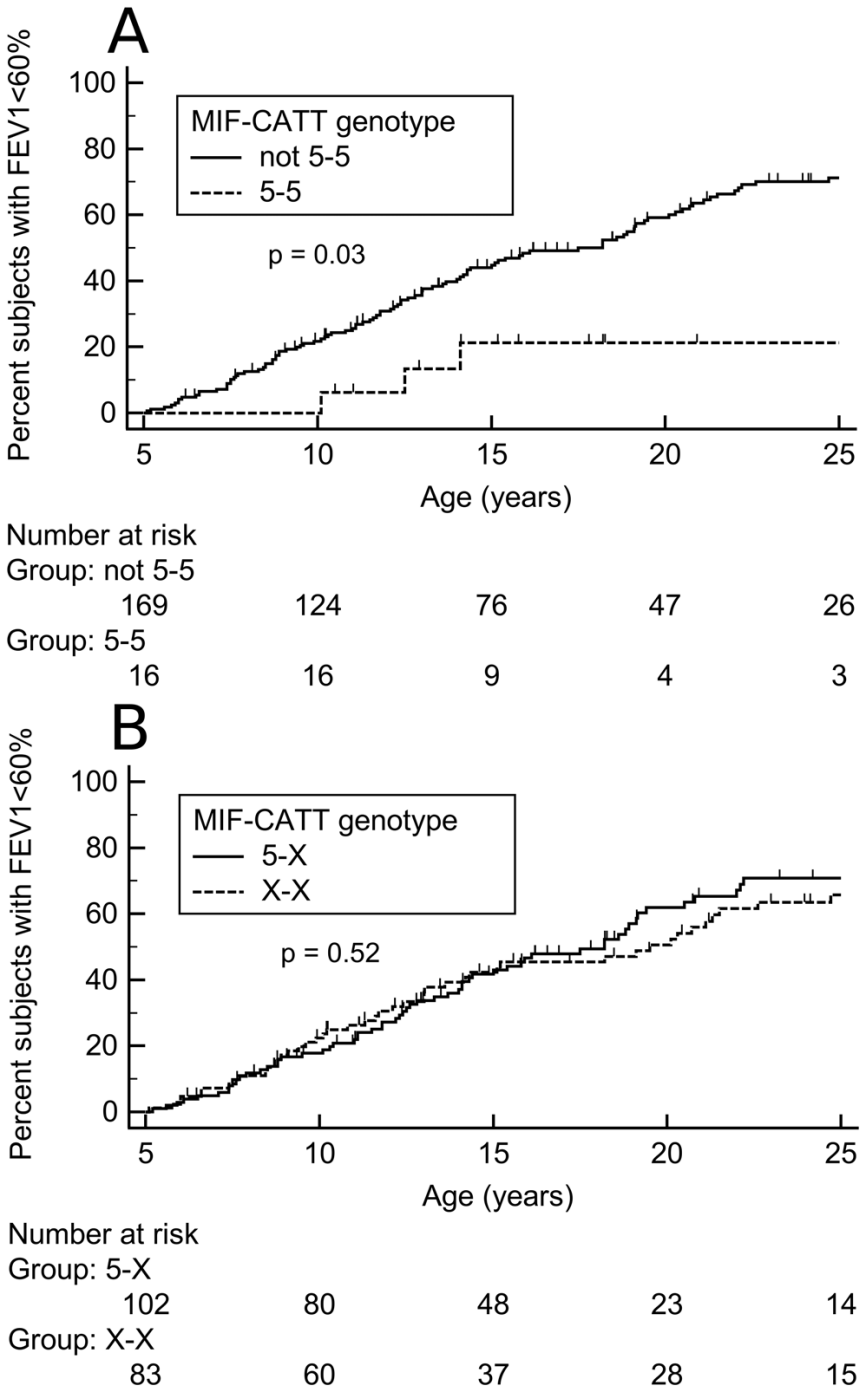
Lung function decline in 185 Cystic Fibrosis patients grouped according to *MIF* -794 CATT genotypes. Kaplan-Meier plots relative to age at first acute episode with FEV1 <60% of predicted value. (A) Comparison between patients with MIF 5-5 (homozygous 5-CATT repeats) vs. not 5-5 genotype; (B) comparison between patients with at least one 5-CATT allele vs. the others. Ticks indicate censored subjects follow-up times. “Number at risk” at the bottom indicates the number of patients without acute episodes at a given time interval and whose follow- up extends at least that far into the curve.

Kaplan-Meier analysis on **Fig. 1** shows that age at first acute episode with FEV1 value below 60% predicted was higher in patients with 5–5 genotype compared to all the other genotypes; median time to event was 29.3 years for 5–5 subjects compared to 18.2 for the others (Hazard ratio = 0.35; 95% CI 0.19–0.66; p = 0.03, **Fig. 1A**). Applying per-allele analysis, no relevant differences emerged, consistent with a recessive effect of 5-CATT allele (**Fig. 1B**).

### MIF-CATT 5-5 genotype shows no association with lower pulmonary function and *P. aeruginosa* chronic colonization under stable conditions

Since recent literature reported that MIF 5-CATT allele also correlates with lower FEV1 under stable conditions and higher prevalence of *P. aeruginosa* colonization [14], [20], we applied multiple linear regression to analyse age-normalized FEV1 [17] under stable conditions, including MIF-CATT 5-5 genotype, patients' origin, chronic colonization by *P. aeruginosa* and presence of insulin-dependent diabetes as covariates. The only factor not associated with FEV1 resulted to be MIF-CATT 5-5 genotype (**table 4**). Variance inflation factor was used to monitor the presence of multicollinearity; its value was below 1.15 for all covariates, indicating no apparent correlation between MIF-CATT genotype and *P. aeruginosa* chronic colonization.

**Table 4:** Multiple regression analysis of FEV1 (Kulich)* data on 189 Cystic Fibrosis patients homozygous for the F508del mutation.

Variable	Coefficient	SE	r-partial	P
Constant	60.96	-	-	-
MIF-CATT genotype = 5-5	7.06	7.09	0.073	0.320
Centre of origin = Brussels	15.15	4.58	0.24	0.001
CC by PA	−16.79	4.16	−0.29	0.0001
Diabetes	−19.04	4.46	−0.30	<0.0001

Overall R^2^ = 0.274; multiple correlation coefficient  = 0.524

Overall significance p-value<0.0001;

FEV1: forced expiratory volume in one second; cc by PA: chronic colonization by *P. aeruginosa*. MIF-CATT genotype: *MIF* gene -CATT repeat genotype at position -794

*CF specific percentile according to Kulich *et al*, *Am J Respir Crit Care Med*, 2005.

## Discussion

In this study, 8% of 189 CF patients homozygous for the F508del *CFTR* mutation carried the 5-5 allele combination for a functional CATT repeat polymorphism in the MIF gene promoter (MIF-CATT), which is expected to be associated with decreased pro-inflammatory activity. Patients carrying this genotype showed a later onset of acute episodes; the rate of lung function decline, as assessed by age at the first FEV1 value below 60% predicted, was lower in this group (table 3). By contrast, no apparent link was found with FEV1 and chronic *P. aeruginosa* colonization under stable conditions at multivariate regression analysis (table 4). Overall, our data support the role of MIF as a modifier gene of lung disease in CF only to a very limited extent, in contrast to what suggested by Plant *et al*. [14] and Adamali *et al*. [20], though the results of both studies differ from ours in several aspects. These authors identified MIF-CATT genotype in 167 adult white CF patients from a single centre, 11% of whom were pancreatic sufficient. Only a fraction of patients (57%) were homozygous for the F508del mutation while 35% were heterozygous for this mutation and the remaining 8% were heterozygous for other *CFTR* mutations. Patients carrying at least one copy of the 5-repeat MIF-CATT allele were found to have a decreased incidence of *P. aeruginosa* colonization (defined as the presence of bacteria in the sputum) and a significant reduction in the risk of pancreatic insufficiency.

The results of Plant *et al.* were partly reproduced (only for FEV and FVC) by Adamali *et al*. in a recent publication of the same research team [20], with a cohort of 143 patients selected from the same referral centre on the basis of a CF diagnosis and not of a F508del genotype. This work presents the same heterogeneous genetic background as in Plant *et al.*, and the same differences compared to our data. Moreover, an unspecified fraction of the patients from this latter study were also enrolled in the former. Interestingly, the *ex-vivo* part of the work by Adamali *et al*. showing differences of MIF levels in plasma and peripheral blood monocytes from CF patients, relies only on individuals with 5-5 and 6-6 MIF genotypes. In these experiments, the 5-5 MIF genotype is confirmed to be the genotype with lowest expression of the protein.

MIF-CATT genotype distribution in patients and controls were comparable in both studies (S2 Table). However, we found that clinical benefit was restricted to later onset of acute episodes in patients homozygous for the 5-repeats MIF-CATT allele.

Aside from these discrepancies, our results are in keeping with the observation by Baugh et al. [13] that in the context of another inflammatory disease (rheumatoid arthritis), only homozygosity for the 5-repeats MIF-CATT allele was protective against the development of severe disease.

Research into CF modifier genes has often yielded conflicting results and numerous challenges have been identified [21]; methodological issues are also likely to be involved. When compared to the earlier study, several strengths of the present work can be stressed. The study population is homogenous at the CF locus, since we focused on a single *CFTR* genotype. The centres involved have been routinely using reliable CF-specific electronic databases for a substantial period of time. Additional efforts were made to normalize data, using CF-specific FEV1 predicted values and multivariate analysis to accout for centre-dependent variations. Z-score for BMI is now considered a more appropriate index expression of nutrition than percentage of ideal body weight [22] and chronic colonization by *P. aeruginosa* is clinically more relevant than airway colonization (simply referring to its presence). A large GWAS study on CF patients failed to test *MIF* as a modifier gene due to the lack of probes for this gene in the used DNA arrays; indeed the whole *MIF* gene is not covered by the Illumina 610-Quad platform used for genotyping. As for possible SNPs in linkage disequilibrium with MIF-CATT, the study used three different cohorts of patients for a total of 3467 CF patients; a sample size that, as the authors themselves stated, is several-fold smaller than the standard for GWAS studies. This implies that only strongest associations might emerge from the study. Due to the small number of MIF 5-CATT subjects and to the fact that, at least from our data, a moderate effect is suggested only in recessive homozygotes, such an effect would have probably been difficult to detect in any case [23]. Altogether the two previously available studies involved an undefined number of CF patients between 167 and 356. This might still be insufficient [24], given the wide range of FEV1 values and the low prevalence of the homozygous 5-CATT *MIF* promoter allele, so further work is encouraged. Besides clinical data, biologic plausibility of a candidate modifier gene is essential and the case of *MIF* gene is a good example. Indeed, it is a key pro-inflammatory mediator that is implicated in the pathogenesis of inflammatory diseases such as asthma, rheumatoid arthritis and acute respiratory distress. It has also been shown to sustain toll-like receptor 4 expression in murine macrophages [25]. Alveolar macrophages are believed to play an important role in regulating the local inflammatory and immune responses in the CF lung [26]–[29]. In addition, both macrophages [30] and their circulating precursors, monocytes [31], have been shown to express functional CFTR, with alveolar macrophages from *CFTR* -/- mice exhibiting defective killing of internalized bacteria [30]. In Cystic Fibrosis, a vicious circle of inflammation and infection leads to destruction of obstructed airways, and it is notable that, up to now, only 4 medications - all with direct or indirect anti-inflammatory properties - are known to slow FEV1 rate of decline in this disease: high-dose ibuprofen [32], inhaled corticosteroids [20], [33], [34], macrolides [35] and DNase [36]. Conceivably, identification of modifier genes of lung disease in CF should help to provide novel therapeutic targets. In this perspective, neutralizing MIF activity by using antibodies or gene knockout, protected mice against severe sepsis by *E. coli* [11] and *Pseudomonas* and also enhanced *P. aeruginosa* airway clearance [10]. Furthermore, several powerful tautomerase inhibitors, highly selective for human MIF, have recently been identified and Adamali *et al*. showed promising *ex-vivo* results regarding their use to tame MIF-sustained immune reaction in CF patients [20], [37], [38]. In conclusion, we have provided additional independent data supporting the role of MIF as a modifier gene of lung disease in CF; the beneficial effect, however, was limited to the homozygous genotype displaying the lowest transcriptional activity. Since the protective MIF genotype is rare, most CF patients could benefit from a targeted approach aimed at reducing the powerful inflammatory response associated with high expression of this gene.

## Supporting Information

S1 Table **Full dataset of Cystic Fibrosis patients used in the present study.**
(XLS)

S2 Table **Comparison of MIF -794 CATT genotype (a) or 5-CAAT allele (b) frequencies between Plant's** [14] **and the present study.**
(DOC)
