# Supplementary material for: Impact of MIF Gene Promoter Polymorphism on F508del Cystic Fibrosis Patients
Source: PLoS One. 2014 Dec 12;9(12):e114274. doi: 10.1371/journal.pone.0114274 (PMC4264759; doi:10.1371/journal.pone.0114274)
Supplement: S2 Table — Comparison of MIF -794 CATT genotype (a) or 5-CAAT allele (b) frequencies between Plant's [14] and the present study. (DOC) [file pone.0114274.s002.doc]

**Supplementary Table S2**: Comparison of MIF -794 CATT genotype (a) or 5-CAAT allele (b) frequencies between Plant’s. and the present study.

a

|  |  | 5-5 | 5-6 | 5-7 | 6-6 | 6-7 | Others* |
| --- | --- | --- | --- | --- | --- | --- | --- |
| Plant (14) | 167 CF (adults) | 5% | 29% | 5% | 49% | 11% | 1% |
|  | 166 Controls | 4% | 30% | 8% | 42% | 12% | 4% |
| Present study | 189 CF | 8% | 38% | 9% | 33% | 11% | 1% |
|  | 135 controls | 9% | 35% | 4% | 36% | 13% | 3% |

* 7-7,7-8 or 8-8 genotypes.

b

|  |  | MIF 5+ | MIF 5- |
| --- | --- | --- | --- |
| Plant (14) | 167 CF | 71 (43%) | 95 (57%) |
|  | 166 controls | 65 (39%) | 102 (61%) |
| Present study | 189 CF | 104 (55%) | 85 (45%) |
|  | 135 controls | 65 (48%) | 70 (52%) |
